# Impact of ‘Double Reduction’ policy on the trend of myopia in school-aged children in Eastern China

**DOI:** 10.7189/jogh.15.04038

**Published:** 2025-02-14

**Authors:** Xiaojun Hu, Weiming Yang, Haodong Ling, Tao Li, Chang Liu, Ruoyan Wei, Zhi Chen, Jiaqi Zhou, Xiaoying Wang, Xiaodong Zhou, Xiaolei Lin, Meiyan Li, Xingtao Zhou

**Affiliations:** 1 Shanghai Institute of Infectious Disease and Biosecurity, Fudan University, Shanghai, China; 2 Eye Institute and Department of Ophthalmology, Eye & ENT Hospital, Fudan University, Shanghai, China; 3 School of Data Science, Fudan University, Shanghai, China; 4 Department of Ophthalmology, Jinshan Hospital of Fudan University, Shanghai, China; 5 NHC Key Laboratory of Myopia (Fudan University); Key Laboratory of Myopia, Chinese Academy of Medical Sciences, Shanghai, China; 6 Shanghai Research Center of Ophthalmology and Optometry, Shanghai, China; 7 Shanghai Engineering Research Center of Laser and Autostereoscopic 3D for Vision Care, Shanghai, China

## Abstract

**Background:**

The high prevalence of myopia among school-aged children has become a significant global challenge. Implementing effective measures, including public policies, is essential to reducing its progression. In this study, we aimed to estimate the impact of the ‘Double Reduction’ policy, introduced in July 2021, on myopia progression among school-aged children in Eastern China.

**Methods:**

We conducted a longitudinal observational study from September 2019 to August 2022 in two cities in Eastern China. The study participants were schoolchildren receiving compulsory education from grades one to nine. We performed a regression discontinuity analysis to calculate the differences in spherical equivalent (SE) and axial length (AL) pre- and post-policy using both community-based and hospital-based cohort data.

**Results:**

Of the included 136 616 participants, 65 903 (48.24%) were girls. The mean age was 10.24 years (standard deviation (SD) = 2.94). Baseline SE averaged −1.47 D (standard error of the mean (SEM) = 0.01), and AL averaged 24.27 mm (SEM = 0.01). After one year of policy implementation, myopic progression was estimated to slow by 0.058 D (95% confidence interval (CI) = 0.043, 0.059) and −0.009 mm (95% CI = −0.05, 0.033) in the right eye within the hospital-based cohort. The community-based cohort showed a similar trend, with a change in SE of 0.115 D (95% CI = 0.017, 0.208) and AL of −0.163mm (95% CI = −0.375, 0.052). The policy had a greater effect on the participants with mild to moderate myopia compared to those with high myopia.

**Conclusions:**

The ‘Double Reduction’ policy slowed the myopia shift at the population level after one year of implementation. A public policy on education reduction might play a role in controlling myopia among schoolchildren, particularly in countries with high myopia prevalence.

Myopia is a common refractive error in which light rays from distant objects focus in front of the retina, resulting in blurred vision. Due to its high prevalence, the condition has emerged as a significant global health issue, particularly in East and Southeast Asia [1–4]. It is associated with an increased risk of sight-threatening diseases and poses considerable medical and socio-economic challenges. These challenges include direct medical costs such as glasses, contact lenses, and corrective surgeries, and indirect costs such as reduced productivity, learning abilities, and quality of life.

The socioeconomic cost of vision impairments was estimated to exceed USD 10 billion in 2021, representing 1.3% of China’s GDP. Myopia also impacts daily life by creating dependencies on glasses or contact lenses and making activities requiring clear-distance vision more difficult. In education, it can impair learning efficiency, classroom participation, and overall academic performance due to eye strain from prolonged reading and screen time. Evidence suggests a strong correlation between the modern rise in myopia and environmental factors [5,6], including excessive education burden [7]. For example, excessive homework and long hours spent on near-work activities and digital screens drastically limit children's outdoor time. Regions with high myopia prevalence often have rigorous education systems, where children face intense academic pressures and parental expectations, even at primary school age [8].

To address this growing public health issue, many nations and organisations have introduced a range of measures aimed at mitigating the growing prevalence of myopia, such as raising public awareness and promoting regular visual screenings [9]. However, these efforts have struggled to control the rapid rise in school-aged myopia due to poor parental compliance [10] and inadequate coordination between education and health ministries to implement measures on a large scale [9]. Effective myopia control strategies should integrate both educational and health aspects, especially as prioritising academic performance over health concerns can undermine public health policies aimed at managing myopia. Consequently, the Chinese government introduced the ‘Double Reduction’ policy in July 2021, targeting elementary and junior high school students (grades one through nine) [11]. This policy seeks to regulate off-campus training, prohibit excessive training, and encourage outdoor activities, promoting a balanced approach to quality education and compulsory education development. It also aims to improve students’ visual health by reducing near-eye activities and increasing time spent outdoors, both of which are crucial for myopia prevention and control. However, consistent implementation across regions and addressing the diverse needs of students remain challenging. The policy has also faced criticisms for its negative impact on academic performance, despite its benefits for student well-being.

There is limited empirical evidence on the effects of nationwide education reduction policies on myopia control. In this longitudinal study, we used data from school-based visual screening cohorts and hospital-based patient cohorts in Eastern China, collected before and one year after the official implementation of the ‘Double Reduction’ policy. We aimed to analyse how the policy influenced myopia progression and incidence, using the regression discontinuity (RD) design to compare spherical equivalent (SE) and axial length (AL) in our sample of schoolchildren. We hypothesised that the ‘Double Reduction’ policy had an impact on myopia prevention and control, with variations in its effects based on students’ age, gender, and baseline myopic status. Our findings could offer insights and recommendations for improving myopia prevention and control among students undergoing compulsory education.

## METHODS

### Patient and public involvement

For this longitudinal observational cohort study, we received approval from the ethics committee of Eye & ENT Hospital, Fudan University. We followed the Declaration in Helsinki in designing in conducting the research: all data in our study were anonymised and written informed consents were signed by all participants who came for eye examinations to approve the use of data for research purposes. For patient and public involvement, we also achieved an agreement with the community representatives during the study design phase and kept the data confidential. All personal identifiers (*e.g*. names, addresses, or ID numbers) were removed or replaced with unique codes before data analysis; we only had access to anonymised data sets.

### Study design

We designed the study within an RD framework, where SE and AL were assumed to be continuously changing with age (*i.e.* time) in case of no ‘Double Reduction’ policy. The SE represents the refractive error, calculated as the sum of the spherical power and half the cylindrical power (*i.e.* SE = sphere + cylinder/2). It is a standardised measure used to quantify myopia or hyperopia. The AL measures the distance from the cornea to the retina, serving as an indicator of myopia progression, as increased AL often correlates with worsening myopia.

We used August 2021 as the point in time at which the ‘Double Reduction’ policy was implemented, and therefore considered September 2019 to July as the pre-policy period and September 2021 to August 2022 as the post-policy period. The ‘Double Reduction’ policy focussed on two key areas: decreasing homework volume and limiting after-school tutoring. It sought to promote a balanced education system, alleviate student stress, and create more equitable learning opportunities by regulating extracurricular academic activities. We used a sharp RD design to estimate the causal impact of the ‘Double Reduction’ policy on SE and AL, with time (September 2019 to August 2022) being the continuous assignment variable and the policy implementation period (August 2021) as the threshold. Specifically, we adopted a linear functional form, which assumes a straight-line relationship between the dependent and independent variables on each side of the cutoff. We tested the validity of the RD design using a placebo test, where we compared causal effects between high school students aged >15 years (not affected by the policy) and non-high school students aged <15 years (affected by the policy). Moreover, the pre-policy time window is crucial for establishing baseline trends in myopia progression for the robustness check. Adjusting this window tests whether the observed effects are robust to variations in the time frame used to estimate pre-policy conditions. If changes in the pre-policy window significantly alter the results, it may indicate that external factors or baseline trends, rather than the policy, influenced the outcomes.

### Study population

We investigated both hospital and community-based cohorts. In the hospital-based cohort, we included participants from the Eye & ENT Hospital, Fudan University with baseline and follow-up outpatient regular refractive examinations. In the community-based cohort, we included participants from the annual school visual screening in ten communities from Shanghai and Changzhou, China. The annual school eye exam dates for the community-based cohort included April to May 2020 (before implementation) and April to May 2022 (after implementation). We used participants aged 6–15 years (before high school) as the treatment group and those aged >15 years (at and post-high school) as the placebo group, since the ‘Double Reduction’ policy was not supposed to affect this population. We excluded participants if they had anisometropia, monocular or binocular amblyopia, or other ocular diseases that might affect refractive measurements.

### Outcomes

Following standardised training, opticians measured noncycloplegic autorefraction (Topcon, Japan) and AL (IOL Master, Zeiss) for participants’ both eyes in the community-based cohort. The opticians in the hospital-based cohort measured the cycloplegic subjective refraction and AL for participants’ both eyes. As they had the necessary expertise, they did not undergo any training. We defined myopia severity as mild (SE = −3, −0.5D), moderate (SE = −6, −3D), and high (SE≤−6D). Here we used SE as the primary endpoint in the analyses, while AL and the severity of myopia were our secondary endpoints.

### Statistical analysis

For descriptive analysis, we presented SE and AL as mean (x̄) and standard deviation (SD), and the prevalence of myopia and myopia severity (mild, moderate, high) as percentages per category. We estimated the causal effect of the ‘Double Reduction’ policy on SE and AL using a RD model for each community cohort as well as for the hospital-based cohort using the following formula:



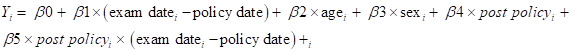



*Y_i_* represents SE or AL for individual *i*, exam date*_i_* represents the date of this eye exam for individual *i*, and post policy*_i_* is an indicator of whether this eye exam occurred before or after the ‘Double Reduction’ policy (post policy = 1 if occurs after policy, post policy = 0 if occurs before policy). In this RD model, the causal effect of the ‘Double Reduction’ policy on SE and AL was given by β4.

Once we estimated the causal effect for each community cohort, we used a Bayesian hierarchical model to combine these causal effects into an average causal effect, where communities with larger sample sizes have a greater impact on the average effect estimate than those with smaller sample sizes (Table S1 in the **Online Supplementary Document**). In addition, we conducted subgroup analyses to investigate the causal effects of the ‘Double Reduction’ policy across different age groups, genders, and different myopic severity categories. We hypothesised that younger children may experience more significant effects from the intervention, as they are more responsive to environmental and behavioural changes. We also hypothesised that boys and girls may respond differently to interventions due to variations in activity patterns or biological factors. We assessed the statistical significance at *P* < 0.05, and we performed all statistical analyses using *R*, version 4.2.1 (R Core Team, Vienna, Austria).

## RESULTS

### Participant characteristics

**Figure 1:**
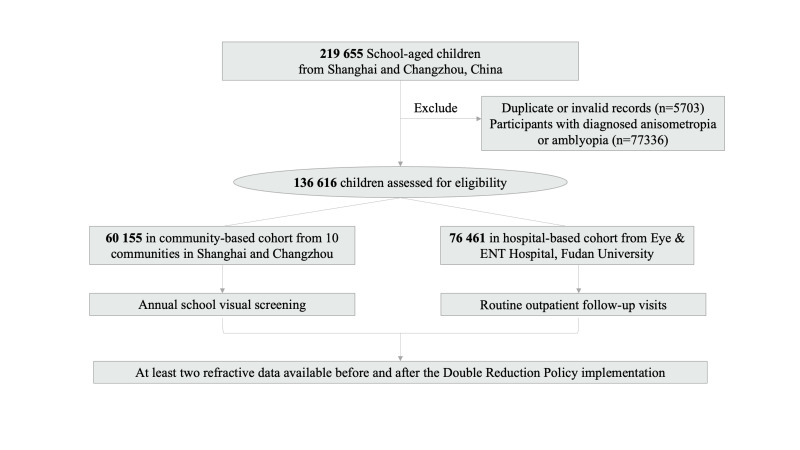
Flow diagram of participants included in this study.

The initial sample consisted of 219 655 participants, including 74 294 participants from ten community-based cohorts and 145 361 from the hospital-based cohort. We excluded 83 039 participants because of anisometropia or amblyopia, resulting in 60 155 community-based and 76 461 hospital-based participants (**Figure 1**). Among them, 48.24% were female, 94.88% were aged between 6 and 15 years, 86.44% had myopia, and only 24.56% had non-missing AL data. The average SE was –1.47 D for the right and –1.78 D for the left eye, while the average AL was 24.47 mm for the right and 24.54 mm for the left eye (**Table 1**). In general, participants in the community-based cohorts tended to have higher SE and shorter AL compared to those in the hospital-based cohort.

**Table 1:** Baseline participant characteristics

	Hospital-based cohort	Community-based cohort
**Total number of participants**	76 461	60 155
**Age in years, x̄ (SD)**	10.26 (2.62)	10.21 (3.28)
**Female, %**	47.78	48.83
**D for right eye SE, x̄ (SE)**	−1.91 (0.01)	−1.62 (0.01)
**D for left eye SE, x̄ (SE)**	−1.45 (0.00)	−1.51 (0.01)
**Myopia severity of right eye, %**		
Mild	58.08	42.43
Moderate	22.49	16.50
High	1.44	4.34
**Myopia severity of left eye, %**		
Mild	86.19	40.89
Moderate	8.61	15.34
High	0.65	4.02
Right AL in mm, x̄ (SE)*	24.24 (0.01)	23.52 (0.01)
**Left AL in mm, x̄ (SE)**	24.20 (0.01)	23.50 (0.01)

AL – axial length, SD – standard deviation, SE – spherical equivalent, SE – standard error, x̄ – mean

*The AL data presented included 8305 participants in the hospital-based cohort and 25 243 participants in the community-based cohort.

### Causal effect estimation under the RD design

**Figure 2:**
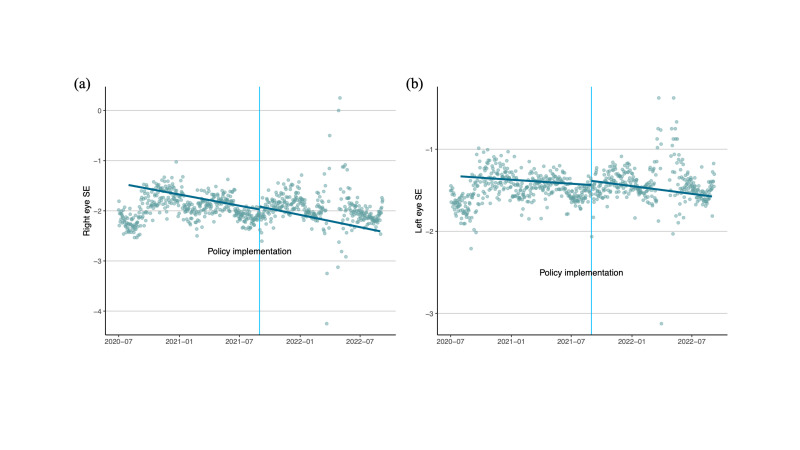
Regression discontinuity analysis for right and left eye SE in a hospital-based cohort. **Panel A.** Right eye SE. **Panel B.** Left eye SE. The dashed lines indicate the ‘Double Reduction’ policy implementation period. SE – spherical equivalent.

For the hospital-based cohort, we noticed a sharp upward jump in SE of both eyes across the discontinuity point (August 2021), indicating a causal impact of the ‘Double Reduction’ policy on SE (**Figure 2**, Panels A and B). According to the RD design and analysis, the ‘Double Reduction’ policy (implemented in August 2021) was predicted to have a causal impact on increasing the left eye SE (slowing down myopia progression) by 0.051 D (95% CI = 0.043, 0.059; *P* < 0.001) and right eye SE by 0.058 D (95% CI = 0.044, 0.072; *P* < 0.001).

**Figure 3:**
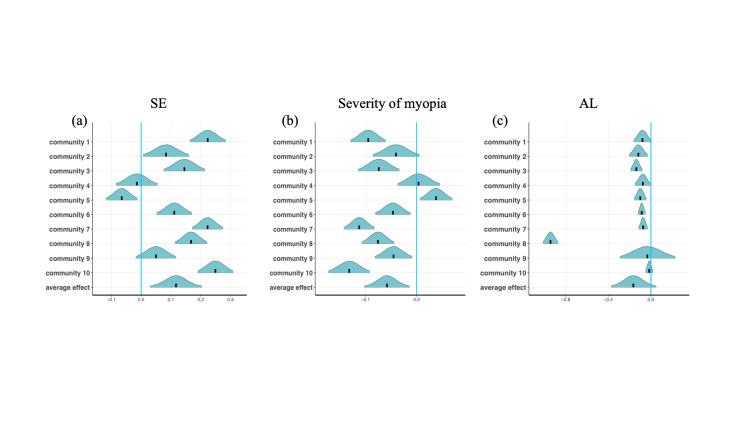
Causal effect of the ‘Double Reduction’ policy estimated for each individual community and the average community-based cohort. **Panel A.** Right eye SE. **Panel B.** Severity of myopia. **Panel C**. AL. AL – axial length, SE – spherical equivalent.

For the community-based cohort, we observed a significant positive causal effect of the ‘Double Reduction’ policy on the right eye SE in six out of the ten communities (**Figure 3**, Panel A), which is confirmed by a negative causal effect of the policy on right eye myopia severity in seven of the ten communities and a negative causal effect of the policy on the right eye AL in eight out of the ten communities (**Figure 3**, Panels B and C). Overall, we observed a significant causal effect of the ‘Double Reduction’ policy in slowing the myopia progression in the right eye. We obtained similar results for the left eye (Figure S1, Panels A–C in the **Online Supplementary Document**).

### Subgroup analysis

We further performed subgroup analysis and evaluated the causal impact of the ‘Double Reduction’ policy on the primary and secondary outcomes across different age groups, genders, and myopia severity categories.

For the hospital-based cohort, we observed the largest causal impact in participants with SE < −3 D for the right eye, compared with SE < −0.5 D (*P* = 0.009) For the left eye, the largest impact was seen in participants with SE < −0.5 D, compared to SE < −3 D (*P* = 0.366) and SE < −6 D (*P* = 0.002) (**Table 2**; Figures S2 and S3 in the **Online Supplementary Document**). For the community-based cohort, we observed the largest causal impact in those with SE < −0.5 D for both eyes, compared with SE < −3 D (*P* = 0.027) and SE < −6 D (*P* = 0.011) for the right eye, and compared with SE < −3 D (*P* = 0.011) and SE < −6 D (*P* = 0.005) for the left eye.

**Table 2:** Causal impact of the ‘Double Reduction’ policy across subgroups with different myopia severity

			Myopia severity*			
	**Mild**		**Moderate**		**High**	
**Cohort**	**Right eye SE**	**Left eye SE**	**Right eye SE**	**Left eye SE**	**Right eye SE**	**Left eye SE**
Hospital-based, x̄ (SE)	−0.006 (0.003)	−0.006 (0.002)	−0.017 (0.004)	−0.005 (0.002)	−0.001 (0.001)	−0.001 (0.000)
Community-based, x̄ (SE)	−0.038 (3.9 × 10^−5^)	−0.035 (3.4 × 10^−5^)	−0.011 (1.6 × 10^−5^)	−0.007 (6.7 × 10^−5^)	−0.002 (7.5 × 10^−6^)	−0.002 (6.8 × 10^−6^)

SE – spherical equivalent, SEM – standard error, x̄ – mean

*We defined myopia severity as mild (SE = −3, −0.5D), moderate (SE = −6, −3D), and high (SE≤−6D).

These results indicate that, in general, the ‘Double Reduction’ policy tends to provide the most protection against myopia progression for students with mild to moderate myopia. The subgroup analysis in both hospital and community-based cohorts further showed that the ‘Double Reduction’ policy had a similar causal effect on SE across age groups and between males and females (Table S1 in the **Online Supplementary Document**).

### Robustness check and placebo test

We tested the robustness of our findings by examining the causal impact over a narrower pre-policy time window. The robustness check revealed minimal variation across different pre-policy time windows, confirming the reliability of the observed effects of policy implementation. Shortening the pre-policy period from July 2020 to September 2022 to August 2020 to September 2022 does not change our main conclusion in the hospital-based cohort, with the estimated causal effect on the left eye SE being 0.096 D (*P* < 0.001) and right eye SE being 0.070 D (*P* < 0.001). However, shortening the pre-policy period did not yield significant results in the community-based cohort due to the lack of data as the community-based eye examination screening was conducted only in the September of each year (Figure S4 in the **Online Supplementary Document**).

We also conducted a placebo test among participants aged between 15–20 years (placebo group), since these high-school students were exempt from the ‘Double Reduction’ policy. In the hospital-based cohort, the causal effect of the ‘Double Reduction’ policy on the left eye SE among the placebo group was estimated to be 0.137 D (*P* = 0.0794), while the effect on the right eye SE was 0.030 D (*P* = 0.5209), meaning it was not statistically significant. We obtained the same results and conclusions from the community-based cohort (Figure S5 in the **Online Supplementary Document**), indicating no causal impact of the ‘Double Reduction’ policy on myopia progression among the placebo group.

## DISCUSSION

In this hospital and community-based cohort study, we found that the ‘Double Reduction’ policy effectively slowed the myopic shift trend among schoolchildren one year after implementation. The overall estimated one-year reduction of SE was approximately 0.05 D, while the decrease of AL was 0.009 mm for the myopic shift in both eyes. While a change of 0.05 D in SE may seem modest, it represents a measurable slowing of myopia progression; even small reductions in annual progression over time can significantly lower the risk of high myopia and associated complications such as retinal detachment or macular degeneration. For younger children, for whom progression rates tend to be faster, this change can meaningfully alter long-term outcomes. The causal effect of the policy was more evident in participants with mild to moderate myopia compared to those with high myopia. This finding supports the hypothesis that reducing the academic burden on schoolchildren might be an effective public policy to slow the current global myopia pandemic, especially in East Asia.

We predicted the trend of myopia progression using two distinct cohorts. The community-based cohort presented a wider, more general population, providing insights into how the policy impacts the broader community. The hospital-based cohort, on the other hand, allowed for more precise and clinically validated measurements of myopia progression, helping confirm findings from the community-based cohort. Additionally, we obtained the refractive data for the community-based cohort using autorefraction, which can sometimes overestimate the actual SE. In contrast, the hospital-based cohort's refractive data were measured by experienced optometrists, ensuring higher accuracy and reliability. By comparing the trends observed in both cohorts, we could validate our findings and ensure their robustness, independent of the measurement method. Furthermore, the hospital-based cohort may have shown smaller changes due to more severe baseline myopia, limiting the potential for noticeable improvement. In contrast, the community-based cohort, representing a broader and potentially healthier population, may have demonstrated more significant effects from the intervention, reflecting the benefits of early detection and management.

We used RD analysis in our study. The ‘Double Reduction’ policy provided a natural experiment with a clear cutoff point, before and after its implementation, making this approach well-suited for identifying causal effects. By comparing children just below and above the threshold, we could credibly assess the policy's impact. The RD design minimised the influence of confounding variables by focussing on a narrow range around the cutoff, assuming children on either side are similar except for their exposure to the policy. This approach isolated the policy's effects from other trends, ensuring that observed changes in myopia progression are attributed to the policy, rather than external factors. These strengths made the RD analysis ideal for evaluating the policy's impact on myopia progression in school-aged children.

Several factors might have contributed to the positive impact of the ‘Double Reduction’ policy on myopia shift. First, the prohibition of excessive academic burden could have resulted in an increased allocation of time to outdoor activities, which was also supported by the fact that the frequency of off-campus activities increased by 75% [12]. Clinical evidence from prior cohort studies and trials validated the assumption that prolonged outdoor time was a protective approach against myopia progression [13,14]. This might be due to the fact that bright light exposure could stimulate the release of dopamine, an important retinal neurotransmitter mediating refractive development, and thus inhibit myopia progression [15,16].

Second, the policy implementation might have raised the public’s awareness of children’s eyesight. Due to their direct interactions, parents play a crucial role in their children’s myopia management. However, existing misconceptions, such as that myopia is a benign condition that could be corrected using optical glass, alongside unawareness of the vision-threatening complications due to an absence of related control, would hinder early intervention [17]. This is confirmed by the findings of a prior study, where nearly two-thirds of rural Chinese students failed to receive appropriate refractive correction in 2008 [18]. However, parent’s attitudes toward such examinations has been changing in recent years [19]. Further educating them on eye care and introducing behavioural interventions, such as text message reminders [20] and school-based health education [21], could also help improve myopia control in this sense.

Third, the policy was found to have led to an increase in sleep duration, with 28.8% of school-aged children reporting longer sleep times [19]. Due to the highly competitive educational environment, 28.9% of schoolchildren slept less than nine hours per day prior to its implementation [22]. Although insufficient sleep duration and poor sleep quality might negatively affect myopia progression [23], they do not appear to be independently associated with myopia, as reported by a cross-sectional study in Singapore [24]. Intrinsic circadian rhythms regulated by melatonin release also play a role in regulating refractive development [25]. As a whole, these factors interact in a complex manner to influence myopia progression. For instance, increased outdoor time not only directly benefits eye health, but can also lead to better sleep patterns and reduced stress, which in turn can influence parental attitudes towards their children's education and well-being. The policy's success in different geographic and cultural contexts will depend on how these factors are prioritised and implemented within those specific societies, considering local attitudes toward education, child welfare, and health.

The policy had a greater impact on myopia progression in children with low to moderate myopia compared to those with high myopia. This may be because the rate of myopic progression is associated with baseline SE [26]. Schoolchildren who were more myopic at baseline tended to progress more rapidly than those with low myopia, reducing the possible protective impact of the education reduction. Similar findings have been observed in previous clinical trials, where increased outdoor time had a larger impact on non-myopic children compared to those with preexisting myopia [14,26,27]. Additionally, studies report that as early as grade three, students experience the greatest decrease in vision, indicating an early onset of poor vision among children in compulsory education [7]. In contrast, interventions may have limited the effectiveness in slowing progression in high myopia cases due to the advanced structural changes in the eye. Furthermore, high myopia may be less influenced by external factors, as its progression is more strongly determined by genetic and physiological factors. Both cohorts showed a similar trend of intervention on myopia progression. These findings emphasise the clinical significance of early intervention to mitigate the risk of developing high myopia in adulthood.

We also observed a trend towards reduced axial elongation that aligned with the slowing myopia progression, though the difference was not statistically significant. This may be due to the smaller sample size of AL data compared to SE data, which was not sufficient to achieve statistical differences. AL is a crucial parameter in myopia research as it directly correlates with the degree of myopia, although it does not have a linear relationship with SE. Additionally, AL measurements were not consistently assessed in some communities due to workplace limitations. Another possible explanation is the asynchronous relationship between AL and SE changes in children during myopia development. Specifically, SE is influenced by both axial elongation and dynamic refractive changes in the lens and cornea. In early childhood, compensatory adjustments in lens thickness or curvature may mask the effects of axial elongation. In contrast, adolescents may experience rapid AL changes with minimal SE variation due to the stabilisation of other refractive components. Previous studies also found differences in SE without corresponding changes in AL [21]. Some studies have shown that axial elongation typically begins three years before myopia onset, while SE changes appear one year later [28]. In addition, axial elongation accelerates before myopia onset and slows significantly once myopia is established [29]. Most participants in our study have developed myopia, which suggests that the deviation of axial elongation may be lower than that observed with SE shift.

Several limitations should be acknowledged in this study. First, the observation period was relatively short, lasting only one year. While the results validated the policy’s significant impact on myopia progression, behavioural changes and their effects on myopia are likely to evolve over a longer timeframe, and a one-year study such as ours might only capture immediate or short-term outcomes rather than long-lasting effects. However, our one year of observation has already demonstrated that the myopia progression could be effectively delayed after the policy, indicating that its nationwide implementation could influence key environmental and behavioural factors contributing to myopia. These findings underscore the importance of timely interventions, such as increasing outdoor activities and reducing academic pressures, in controlling myopia progression. Our findings might, therefore, provide insights for governments and professionals when promoting large-scale myopia control measures, particularly in regions with a high myopia prevalence. Further long-term follow-up studies are necessary to assess the sustained impact of the policy.

Second, the community-based cohort did not undergo cycloplegic refraction due to the limitations in school vision screening, which may have led to an overestimation of myopia incidence or progression. However, our primary objective of comparing myopia trends before and after the policy implementation ensured the robustness of the conclusions. Additionally, the hospital-based cohort used cycloplegic and subjective refractive data, which are highly accurate and yield comparable results. Despite these limitations, our study has several strengths, including a robust study design and data collection from a large sample size of school-aged children across multiple schools and regions. Furthermore, we excluded many participants from the initial cohort (*e.g.* participants with anisometropia or amblyopia) to ensure data consistency and analytical validity. We also did so in the community-based cohort due to measurement. While these exclusions reduced the overall sample size, they resulted in a more homogenous cohort.

## CONCLUSIONS

The nationwide ‘Double Reduction’ policy implementation effectively slowed the myopia progression trend by alleviating the academic burden among school-aged children receiving compulsory education. Continued collaboration between educators, policymakers, and eye care professionals is essential in exploring the impact of reduced academic pressure on myopia prevention and developing public policies on vision health in educational settings, especially in East Asia.

## Additional material


Online Supplementary Document
