# Supplementary material for: Impact of ‘Double Reduction’ policy on the trend of myopia in school-aged children in Eastern China
Source: J Glob Health. 2025 Feb 14;15:04038. doi: 10.7189/jogh.15.04038 (PMC11825120; doi:10.7189/jogh.15.04038)
Supplement: Online Supplementary Document [file jogh-15-04038-s001.pdf]

Supplement to: Hu X, Yang W, Ling H, Li T, Liu C, Wei R, Chen Z, Zhou J, Wang X, Zhou X, Lin X, Li M, Zhou X. Impact of 'Double Reduction' policy on the trend of myopia in school-aged children in Eastern China. J Glob Health. 2025;15:04038.

Table S1: Causal impact of the "Double Reduction" Policy across subgroups with different age and sex.

		age 6-10y, mean (SEM)	age 11-15y, mean (SEM)
local causal effect in Hospital-based cohort	right eye SE	0.057 (0.009)	0.067 (0.011)
	left eye SE	0.050 (0.005)	0.071 (0.008)
estimated causal effect in community-based cohort	right eye SE	0.136 (1.1×10^{-4})	0.073 (1.2×10^{-4})
	left eye SE	0.104 (9.4×10^{-5})	0.089 (1.2×10^{-4})

		female, mean (SEM)	male, mean (SEM)
local causal effect in Hospital-based cohort	right eye SE	0.050 (0.010)	0.068 (0.010)
	left eye SE	0.046 (0.006)	0.056 (0.006)
estimated causal effect in community-based cohort	right eye SE	0.113 (1.2×10^{-4})	0.118 (1.0×10^{-4})
	left eye SE	0.089 (1.0×10^{-4})	0.109 (9.4×10^{-5})

The subgroup analyses revealed that for children aged 6-10 years, the local causal effect in the hospital-based cohort was 0.057 (0.009) for the right eye and 0.050 (0.005) for the left eye. In the 11-15 year age group, these effects were slightly higher at 0.067 (0.011) for the right eye and 0.071 (0.008) for the left eye. Community-based cohort estimates showed a right eye SE effect of 0.136 and a left eye SE effect of 0.104 for the 6-10 age group, with 0.073 and 0.089 respectively for the 11-15 age group.

The gender-based analysis demonstrated a consistent effect of the policy, with females experiencing a local causal effect of 0.050 (0.010) for the right eye and 0.046 (0.006) for the left eye in the hospital-based cohort, and males showing 0.068 (0.010) for the right eye and 0.056 (0.006) for the left eye. In the community-based cohort, the estimated causal effects were 0.113 for females and 0.118 for males in the right eye SE, with 0.089 and 0.109 for the left eye SE, respectively.

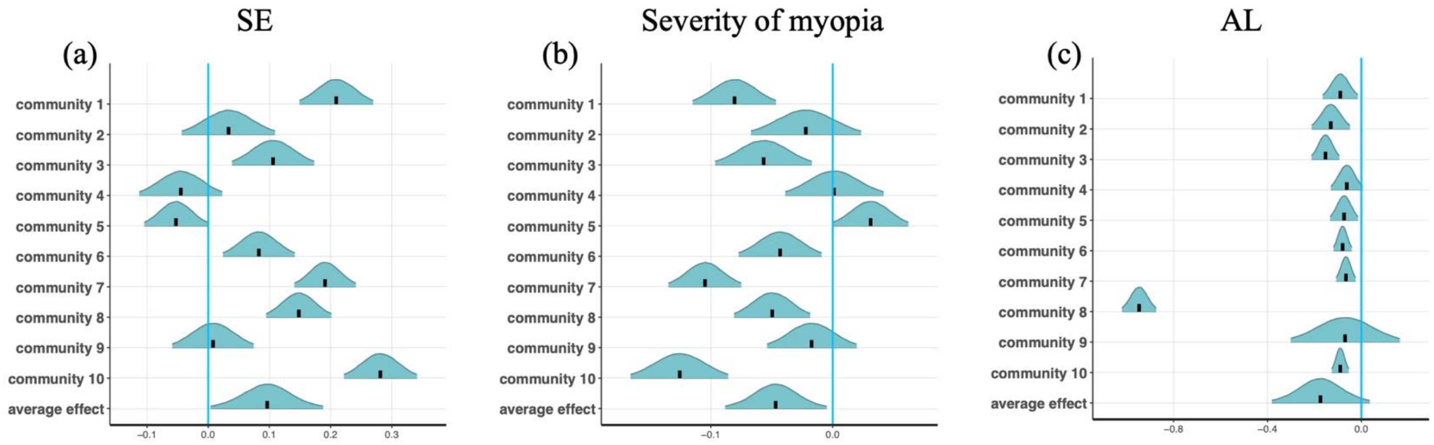

Figure S1: Causal effect of the "Double Reduction" Policy on left eye spherical equivalent (SE) (a), the severity of myopia (b) and axial length (AL) estimated for each individual community as well as for the average community-based cohort. Filled area under the curve represents 95% confidence interval.

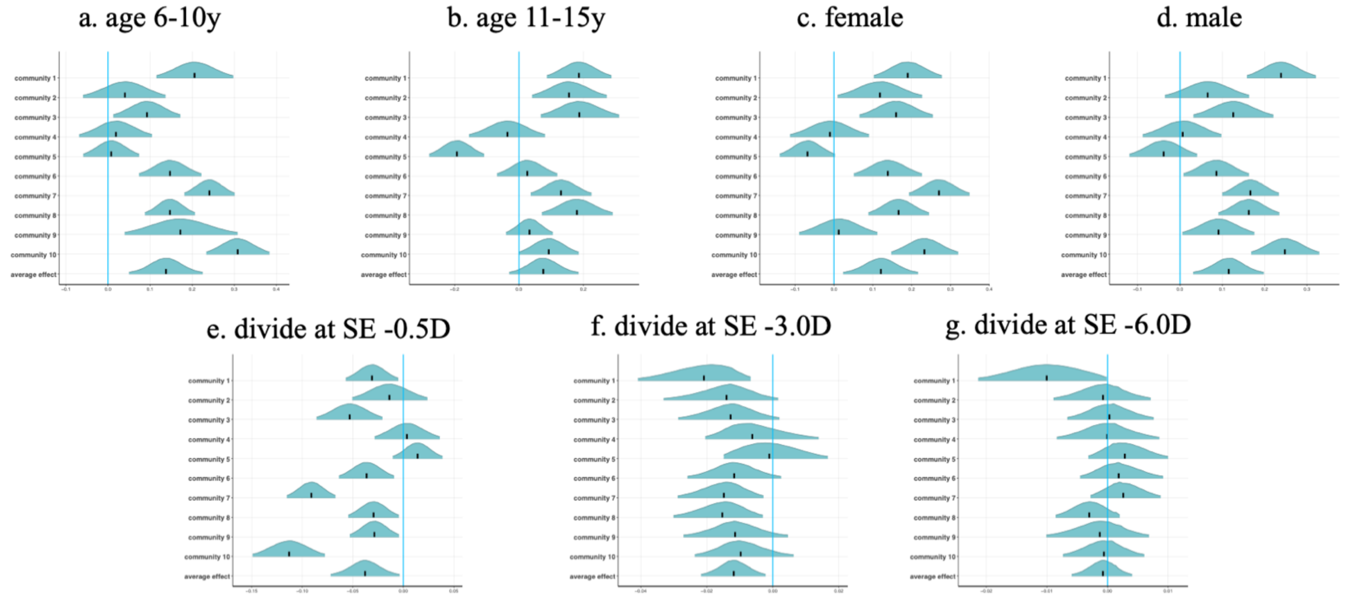

Figure S2: Estimation of “Double Reduction” Policy’s local causal effect on right eye SE in subgroups of a community-based cohort.

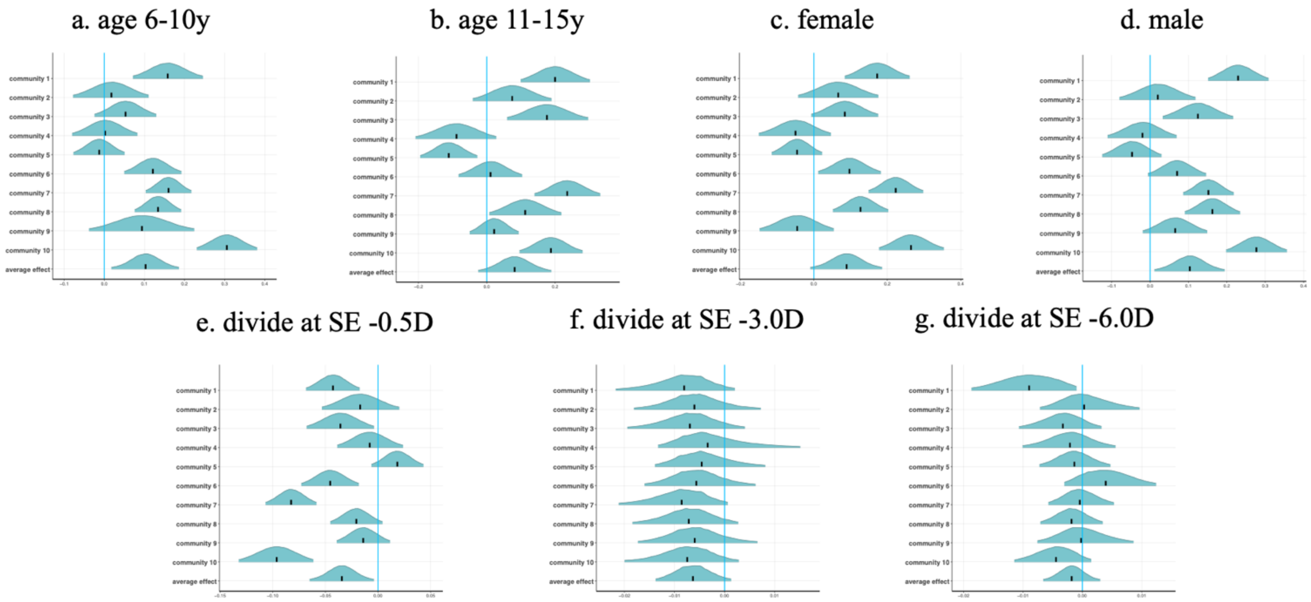

Figure S3: Estimation of “Double Reduction” Policy’s local causal effect on left eye SE in subgroups of a community-based cohort.

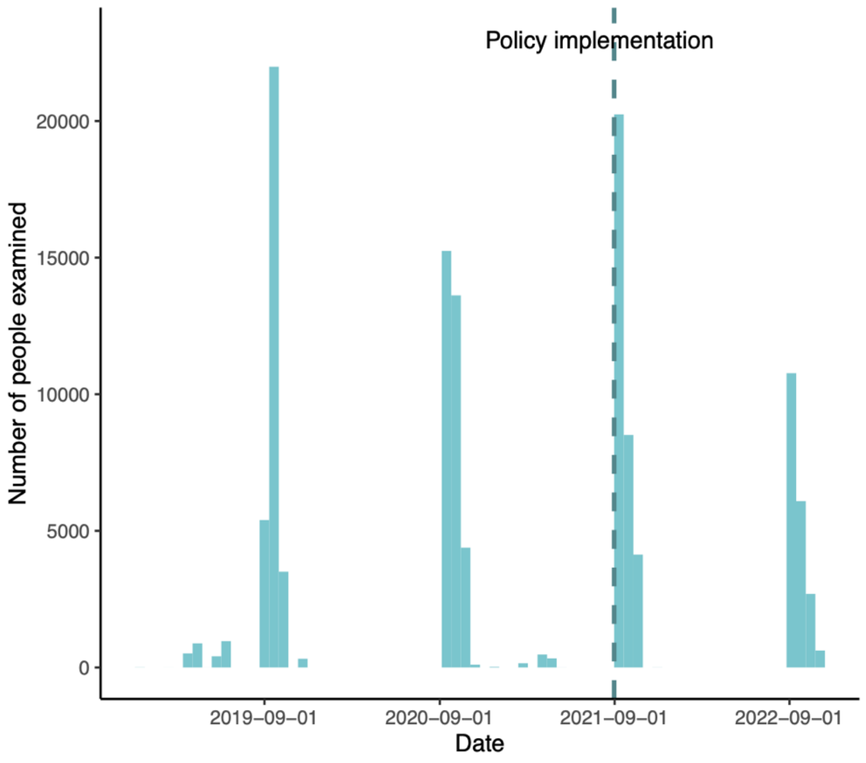

Figure S4: Eye examination dates in communities. The dashed lines indicate the “Double Reduction” Policy implementation period.

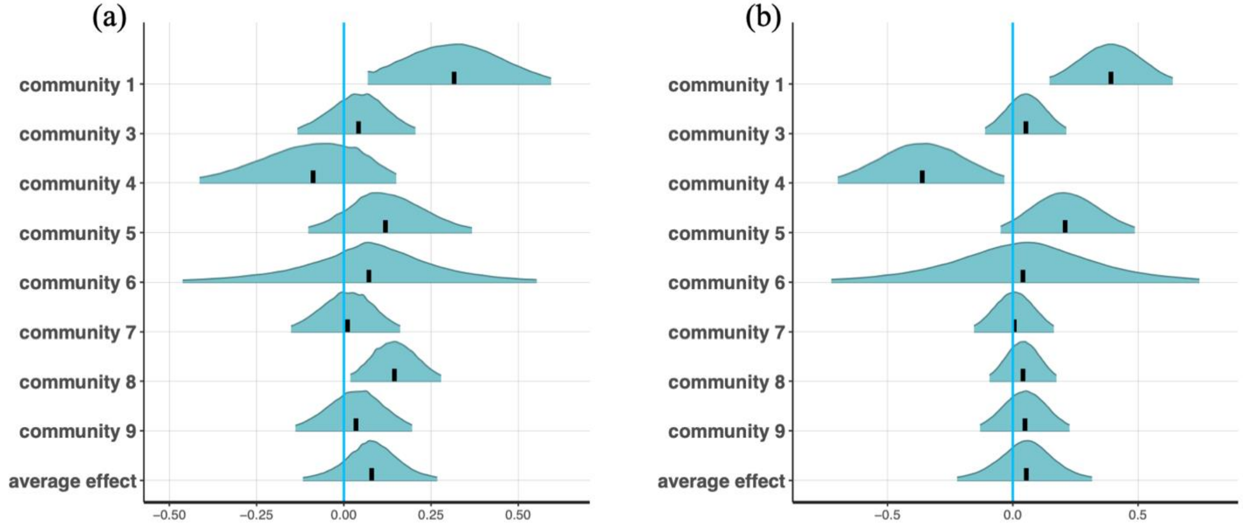

Figure S5: Causal effect of the "Double Reduction" Policy on right eye spherical equivalent (SE) (a) and left eye SE (b) estimated for participants aged 15 to 20 in each individual community as well as for the average community-based cohort. Community 2 and 10 are not included due to insufficient data. Filled area under the curve represents 95% confidence interval.
